# Postural Control Follows a Bi-Phasic Alteration Pattern During Mountain Ultra-Marathon

**DOI:** 10.3389/fphys.2018.01971

**Published:** 2019-01-17

**Authors:** Francis Degache, Emilie Serain, Gianluca Vernillo, Frederic Meyer, Mathieu Falbriard, Aldo Savoldelli, Kenny Guex, Grégoire P. Millet

**Affiliations:** ^1^School of Health Sciences, University of Applied Science and Arts Western Switzerland, Lausanne, Switzerland; ^2^Human Performance Laboratory, Faculty of Kinesiology, University of Calgary, Calgary, AB, Canada; ^3^Research Center for Sport, Mountain and Health (CeRiSM), University of Verona, Rovereto, Italy; ^4^Institute of Sports Sciences, Faculty of Biology and Medicine, University of Lausanne, Lausanne, Switzerland; ^5^Laboratory of Movement Analysis and Measurement, STI, Ecole Polytechnique Federale de Lausanne (EPFL), Lausanne, Switzerland; ^6^Department of Neurosciences, Biomedicine and Movement Sciences, University of Verona, Verona, Italy

**Keywords:** postural control, balance, altitude, ultra-endurance, compensatory strategies, fatigue

## Abstract

It is well knows that postural control (PC) is deteriorated with neuromuscular fatigue, altitude or sleep deprivation induced by a mountain ultra-marathon (MUM). Several regulatory mechanisms have also been reported during this type of event and the changes in PC at different points of MUM remain unknown. The purpose of this study was to investigate the time course of PC during an extreme MUM. We tested the hypothesis that PC alteration would not increase linearly.

**Methods:** 16 participants (age 45.1 ± 9.6 years) were tested bipedaly on a posturographic platform for 51.2 s with eyes open every ∼50 km. Both traditional and stabilogram diffusion analyses (SDA) were performed. A visual analog scale (VAS) was used for a subjective evaluation of global fatigue, sleep feeling and pain.

**Results:** The main parameters (center of pressure trajectory analysis) increased significantly (*p* < 0.001, *d* = 1.56, very large) until km 100. This was confirmed by SDA in the antero-posterior plane. Short term effective diffusion coefficient significantly increased (*p* < 0.001, *d* = 1.07, very large) as critical point (*p* < 0.01, *d* = 1.57, very large). From km 100 to 200, a different response was observed with a continuous decrease in most of the PC parameters. This was confirmed by SDA in the antero-posterior plane. Short term effective diffusion coefficient significantly increased (*p* < 0.001, *d* = 1.39, very large) as critical point (*p* < 0.01, *d* = 1.51, very large).

**Conclusion:** Posture alteration is progressively increased until 100 km. After this point, compensatory mechanisms appear to limit the posture degradation. This bi-phasic response is of interest for better understanding the coping with extreme fatigue.

## Introduction

Mountain Ultra-Marathon (MUM) races have become more and more popular in recent years (Millet et al., 2011; Millet and Millet, 2012; Saugy et al., 2013; Vernillo et al., 2015a; Degache et al., 2016; Zakovska et al., 2017). These events involve a distance longer than traditional marathons on mountain trails (Millet and Millet, 2012). Studies of this type of extreme race are an opportunity to investigate the physiological impact on the human body (Millet and Millet, 2012; Zakovska et al., 2017; Knechtle and Nikolaidis, 2018). One of them, the Tor des Geants (T*dG*), is considered as one of the world’s most challenging single-stage MUM and it was the support of previous studies from our group investigating the alterations of pulmonary (Vernillo et al., 2015a), energetic/biomechanics (Vernillo et al., 2015b; Degache et al., 2016), and neuromuscular function (Saugy et al., 2013) as well as postural control (PC) (Degache et al., 2014). Particularly, Degache et al. (2014) reported a significant alteration of PC after the T*d*G, that is the runners took more time to stabilize their body post-race. Postural stability can be affected by sleep deprivation, but this last study showed minimal alterations in balance in controls with the same level of sleep deprivation, that’s why their conclusion was that the postural alterations originated predominantly from the MUM characteristics, (e.g., altitude, running uphill/downhill) and from muscle fatigue, and not from sleep deprivation itself (Degache et al., 2014).

PC is a complex function, as it relates to the fact that the human body is a multi-segment biomechanical chain: dynamic interaction between body segments and movements is required in order to produce joint torque corrections in all the joints (Alexandrov and Frolov, 2011). By maintaining the projection of the center of mass (C*o*M) within the base of support, the sensory system, including complex interaction between the somatosensory, vestibular and visual sources, contributes to PC. By using a posturographic platform, analyzing center of pressure (C*o*P) trajectory can be recorded and permit to analyze the PC parameters (Winter et al., 1996; Caron et al., 2000). A more recent conceptual framework for studying human PC was developed and applied to analyze PC parameters with stabilogram diffusion analysis (SDA) (Collins and Luca, 1992; Peterka, 2000). In this approach, the C*o*P is modelized as fractional Brownian motion, which is characterized by the effective stochastic activity of two PC systems during quiet standing: a short-term and long-term mechanism during first 10 s of bipedal standing test. Parameters of SDA suggest that during short-term intervals, open-loop control mechanisms are utilized, whereas during long-term intervals, closed-loop mechanisms are presented (Collins and Luca, 1992; Peterka, 2000). The transition between these two types of control is termed the critical point. This transition point is defined either the critical time intervals (Ct = Δ*t*_c_) and or the critical mean square displacement (Cd = Δ*j*^2^) and quantify the spatial and temporal characteristics of the switching mechanisms.

Recent research on MUM showed that loss of strength does not linearly increase with the exercise duration (Saugy et al., 2013). It was suggested that such ultra-distance exercise seems to induce relative anticipatory strategies and adaptive responses to extreme load (Saugy et al., 2013; Degache et al., 2016). Another protective mechanism was found about the running biomechanics during the T*d*G, it was demonstrated that the runners modified their running patterns during the first half of the race to switch to a safer and smoother technique to minimize pain of the eccentric phase and to anticipate further muscles damages (Degache et al., 2016). There is no literature about how PC evolve through MUM and because this kind of event are more popular it seems important to understand the underlying mechanism of the body in order to prevent injuries.

The main purpose of this study was to investigate how the PC evolved throughout the T*d*G. In relation with previous studies on the T*d*G (Saugy et al., 2013; Degache et al., 2014; Vernillo et al., 2015a,b; Degache et al., 2016) and the fact that anticipatory strategy appears in this type of extreme event, we tested the hypothesis that the time course of PC would not increase linearly.

## Materials and Methods

Sixteen male (aged 45.1 ± 9.6 years, heighted 1.78 ± 0.09 m and weighed 75.6 ± 8.6 kg) participated in this study. All were volunteers, trained and experienced an in ultra-marathon and trail running (third participation on TdG for 4 runners).

All the participants were fully informed of the procedure and the risks involved. They all voluntary provided written an informed consent prior the participation on the study and they were allowed to drop-out from the study at will. This study was approved by the institutional ethics committee of the University of Verona, Italy (Approval #152, Department of Neurological, Neuropsychological, Morphological and Motor Sciences). The experiment was conducted according to the Declaration of Helsinki.

**FIGURE 1:**
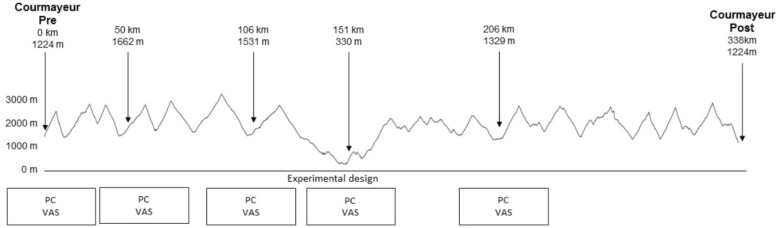
GPS profile of the total race with the test locations and the experimental design (PC: postural control, VAS: visual analog scale).

The race supporting this study was the 6th edition of T*dG*, held on 2015 (Figure 1), with 474 participants at the start of the race. It consists of running/walking 338.6 km with a total of 30,914 m positive and negative elevation change. The altitude along the course ranges between 3300 and 322 m, with 20 mountain passes over 2000 m. The maximum time allowed for completion of the race is 150 h. The distance is divided into seven stages with six aid-stations every 50-km where runners can rest and sleep. The participants can pace themselves and manage their stop as they wish, as the recovery stop is not subtracted from the race time. There were no familiarization sessions and the runners were tested at the start and four times at different aid stations (Figure 1). Because of exceptional inclement weather, the race was stopped at the 206th km (Table 1). The average race time was 73.14 ± 8.5 h, (73 h, 8 m, 24 s ± 8 h, 30 m).

A posturographic platform (Fusyo-Medicapteur, Toulouse, France) with the Win-posturo software (2.4 Medicapteur, Balma, France) was used to calculate C*o*P displacement. The posturographic platform, equipped with three pressures gauges (hysteresis < 0.2%), measured 530 × 460 × 35 mm and the sampling rate was 40 Hz. The test duration was 51.2 s, resulting in 2048 points time series. The participants were placed on precise markers and their feet formed a 30° angle. They were instructed to stand double leg with their arm at their sides, to fix a target at a distance of 1 m, and to keep their eyes open (EO). Data was collected every ∼50 km by using this posturographic platform.

Participants were requested to quantify their level of general feeling (subjective general fatigue, sleep feeling) and subjective pain in three anatomical areas (foot-ankle, leg-knee; thigh-hip) by using the visual analog scale (VAS) (Tseng et al., 2010; Saugy et al., 2013). They were asked to mark the general feeling and their pain level on the VAS with a 100 mm horizontal line with “no fatigue/no pain/no sleepy” on one end (0 mm) and “extremely fatigued/painful/sleepy” on the other (100 mm).

C*o*P data were collected to extract the standard postural sway parameters. For each participants, the following variables were computed: (1) Surface of *Co*Ps (mm^2^) (90% confidence ellipse); (2) Total displacement of C*o*Pxy in anteroposterior (AP) and mediolateral (ML) (total length in mm); (3) ML displacement of C*o*Px (mm); (4) AP displacement of C*o*Py (mm); and (5) Mean speed of C*o*Pms, (mm s^−1^). The mean speed represent the total displacement divided by the sampling time.

In addition, SDA was performed for each subject, each recording time and each condition. The SDA summarize the mean square of C*o*P displacement as a function of the time interval between C*o*P comparisons (Collins and Luca, 1992).

This analysis was repeated for time intervals ranging from 1/40 to 10 s. The SDA parameters were extracted with Collins and Luca (1992) routine and the following parameters were taken for the ML(x) and AP (y) (Collins and Luca, 1992):

(1) The linear regression of the diffusion coefficients for short- and long-term region (D*s*, D*l*) (mm^2^ s^−1^); (2) The point of intersection between the short- and long-term regions of the linear–linear plot is the critical point *C* (Collins and Luca, 1992). The coordinates for the critical point (*Ct*, *Cd*) provide the measures of the *critical time interval*, i.e., Ct = Δ*t*_c_, and *critical value*, Cd = Δ*j*^2^.

Running speed (km h^−1^) for each running section (Sections 1–4) was calculated with a flat-equivalent distance with the following formula (Saugy et al., 2013):

$$\begin{array}{c} \mathrm{Flat}-\mathrm{equivalent distance}=\\ \;\mathrm{distance}\;(\mathrm{km})+\mathrm{positive elevation change}\;(m)/100 \end{array}$$

For VAS of pain, the mean of right and left side was set together to represent a global value of subjective values for pain. The mean value of VAS for general feeling (global fatigue, sleep feeling) was used.

For all the data mean value ± standard deviation (SD) are presented.

The normality of the samples was checked with the Shapiro–Wilk test. To compare each dependent variable between all conditions, a one-way repeated measures analysis of variance (ANOVA) was used. Tukey *post hoc* tests were used to localize the differences between means.

Pearson correlations coefficient between the standards parameters of PC, all subjective values of the VAS, the flat equivalent speed was tested with delta values (%) for all conditions.

We added effects size analyze for all variables (Table 2B).

For all statistical analysis, the software Sigmaplot (Version 12.5; Systat Software Inc., San Jose, CA, United States) was used. A *p* value of ≤0.05 was accepted as the level of significance.

## Results

**FIGURE 2:**
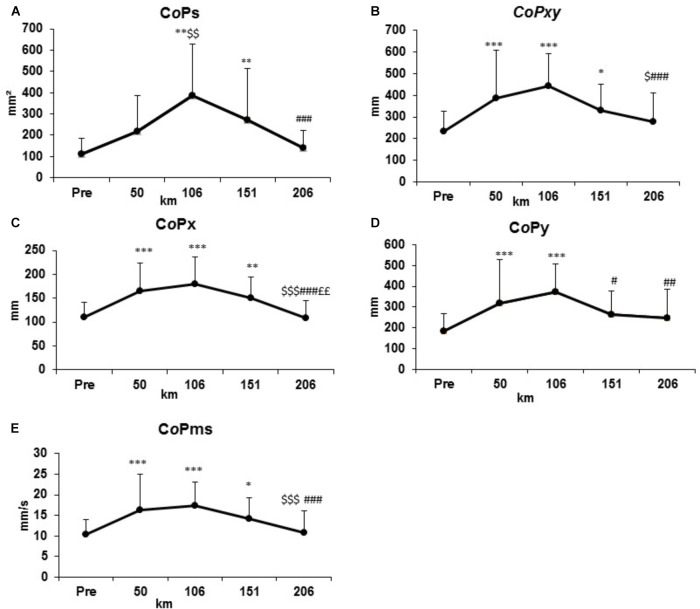
Evolutions of standard postural control parameters represented by surface (C*o*Ps, **A**), total displacement (C*o*Pxy, **B**), ML displacement (C*o*Px, **D**), AP displacement (C*o*Py, **C**), mean speed (C*o*Pms, **E**) at the condition PRE and every 50 km.

The evolution of standard parameters of PC is shown in Figure 2 and values are presented in Table 1A. All effect size are presented in Table 1B. At km 50, the C*o*Pxy, C*o*Px, C*o*Py, and C*o*Pms significantly increased compared to PRE condition (*p* < 0.001 for all of these parameters) panel B–E). At km 106, the same parameters and the C*o*Ps increased significantly compared to PRE condition (*p* < 0.01) (Panels A–E). At km 151, C*o*Ps, C*o*Pxy, C*o*Px, C*o*Pms were significantly different from the condition PRE (*p* < 0.05) (Panels A, B, D, E). Only C*o*Py was statistically different from km 106 (*p* < 0.05) (Panel C). In the last section, all parameters are statistically different from km 106 (*p* < 0.01) without a significant difference with the PRE condition (Panels A–E).

**Table 1A:** Results (mean ± SD) of standard postural control (PC) parameters represented by surface (*Co*Ps), total displacement (C*o*Pxy), Anteroposterior displacement (C*o*Py), mediolateral displacement (C*o*Px), mean speed (C*o*Pms) during the condition PRE and every 50 km.

	PRE	50 km	106 km	151 km	206 km
*CoPs* (mm^2^)	10.43 ± 3.55	16.22 ± 8.82	17.34 ± 5.83^∗∗∗$$^	14.23 ± 4.99^∗∗^	10.84 ± 5.29^###^
*CoPxy* (mm)	235.55 ± 91.48	387.49 ± 219.36^∗∗∗^	443.92 ± 149.34^∗∗∗^	331.64 ± 117.96^∗##^	277.5 ± 135.34^###^
*CoPx* (mm)	110.21 ± 31.94	164.46 ± 59.17^∗∗∗^	179.16 ± 57.37^∗∗∗^	149.62 ± 44.19^∗∗^	108.57 ± 35.69^###$$$^
*CoPy* (mm)	184.27 ± 84.43	316.71 ± 209.56^∗∗∗^	371.96 ± 134.18^∗∗∗^	265.03 ± 110.57^#^	247.41 ± 139.23^##^
*CoPms* (mm s^−1^)	10.43 ± 3.55	16.22 ± 8.82^∗∗∗^	17.34 ± 5.83^∗∗∗^	14.23 ± 4.99^∗^	10.84 ± 5.29^###$$$^

*^∗^p < 0.05, ^∗∗^p < 0.01, ^∗∗∗^p < 0.001 for differences compared to PRE, ^$$^p < 0.01, ^$$$^p < 0.001 for differences compared to 50 km, ^#^p < 0.05, ^#^p < 0.05, ^##^p < 0.01, ^###^p < 0.001 for differences compared to 106 km.*

**Table 1B:** Effect size of classical PC parameters.

	Pre-50 km effect size	Pre-106 km effect size	Pre-151 km effect size	50–106 km effect size	106–151 km effect size	50–206 km effect size	106–206 km effect size
*CoPs* (mm^2^)	–	1.55 Very large	0.91 Large	0.88 Large	–	–	1.37 Very large
							
*CoPxy* (mm)	0.91 Large	1.68 Very large	0.91 Large	0.83 Large	–	–	1.16 Very large
							
*CoPx* (mm)	1.14 Very large	1.48 Very large	1.02 Very large	–	–	1.14 Very large	1.47 Very large
							
*CoPy* (mm)	0.57 Medium	1.67 Very large	–	–	0.87 Large	–	0.91 Large
*CoPms* (mm s^−1^)	0.86 Large	1.43 Very large	0.87 Large	–	–	0.74 Medium	1.17 Very large

The same kinetic of changes in the SDA parameters was observed. In the AP plan (Table 2), D*sy* showed a significantly greater short-term effective stabilogram diffusion at km 50 (*p* < 0.01) and 106 (*p* < 0.001) compare to PRE, with a peak value at km 106. From that point, D*sy* were not statistically different with the PRE condition, but they were statistically different with the km 106 (*p* < 0.05). The short-term region in the ML plane (D*sx*) showed a significant difference at km 106 with the PRE condition (*p* < 0.01) and km 206 with km 106 (*p* < 0.01).

**Table 2:** Results of SDA Parameters in the AP plan with short term effective diffusion coefficient (D*sy*), long term effective diffusion coefficient (D*ly*), critical point (C*t*, C*d*) during the condition PRE and every 50 km.

	PRE	50 km	106 km	151 km	206 km
D*SY* (mm^2^ s^−1^)	5.75 ± 4.15	19.94 ± 25.89^∗∗^	28.78 ± 22.07^∗∗∗^	12.70 ± 10.34^#^	6.48 ± 4.97^###^
D*LY* (mm^2^ s^−1^)	1.01 ± 1.89	1.91 ± 1.29	3.80 ± 5.89	1.52 ± 1.81	0.87 ± 1.03
C*TY* (Δ*t*_c_)	1.26 ± 1.91	0.30 ± 0.68	1.63 ± 1.57	0.93 ± 1.59	0.63 ± 3.47
C*DY*(Δ*j*^2^)	8.57 ± 18.60	13.18 ± 17.78	58.35 ± 40.66^∗∗∗$$^	20.27 ± 23.80^###^	9.91 ± 20.07^###^

*^∗∗^p < 0.01, ^∗∗∗^p < 0.001 for differences compared to PRE, ^$$^p < 0.01 for differences compared to 50 km, ^#^p < 0.05, ^##^p < 0.01, ^###^p < 0.001 for differences compared to 106 km.*

In contrast, the long-term region effective diffusion, represented by the D*lx, Dly*, did not change significantly during the race in AP and ML plane (Table 2).

The critical displacement (C*d*y) in the AP was significantly higher at km 106 compare to PRE condition (*p* < 0.001) and km 50 (*p* < 0.01) (Table 2). C*d*y values at km 151 and 206 were not statistically different from PRE condition but were different with km 106 (*p* < 0.001). In the ML plane, C*dx* showed no significant results. The critical time (C*t*) in both directions (AP, ML) did not change in runners during the race.

**FIGURE 3:**
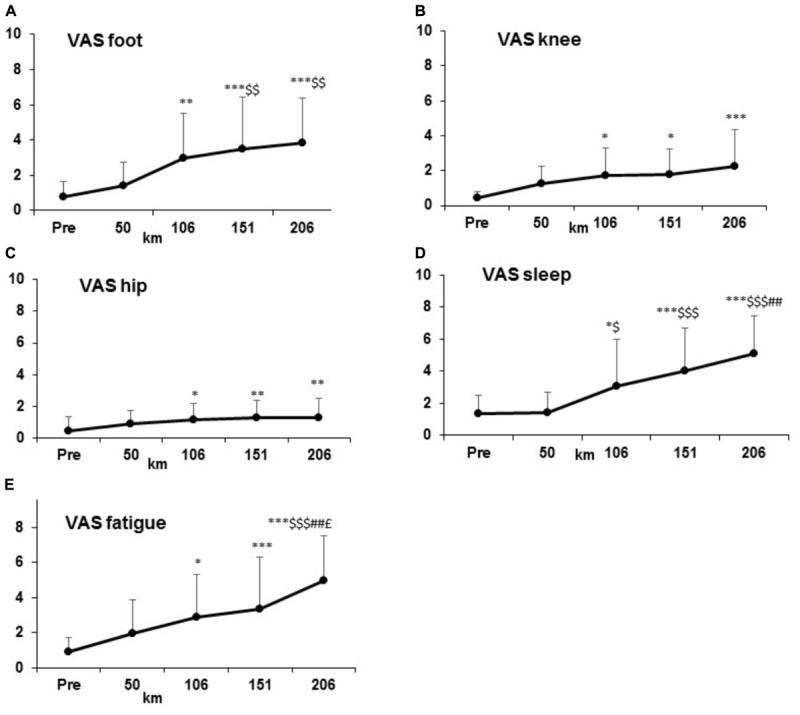
Increase of Visual Analogic Scale (VAS) parameters: subjective pain values **(A–C)**, general feeling **(D–E)** at condition PRE and every 50 km.

In general, all parameters (perceived level of fatigue, sleep feeling, and pain increased) increased significantly form km 106 (*p* < 0.001) and linearly until km 206 (*p* < 0.001) (Figure 3).

Race time, running speed, equivalent flat distance, positive elevation (D+) and time during each section are presented in Table 3. Initial running speed (flat speed equivalent), of 8.4 ± 1.1 km h^−1^, decreased significantly during the different section to reach a walking speed of 5.1 ± 0.9 km h^−1^ in the last section (*p* < 0.001).

**Table 3:** Technical characteristic of the MUM.

	Pre-50 km *Section1*	50–106 km *Section2*	106–151 km *Section3*	151–206 km *Section4*
Race time (h)	11.77 ± 1.54	18.77 ± 2.59	12.10 ± 1.78	23.24 ± 3.79
Speed (flat speed equivalent - km h^−1^)	8.41 ± 1.10	5.90 ± 0.79^∗∗∗^	6.10 ± 1.09^∗∗∗^	5.09 ± 0.93^∗∗∗$$$$$^
Equivalent flat distance (km)	97.5	108.8	72.0	114.9
D+ (m)	4747	5082	2698	6086

*^∗∗∗^p < 0.001 for difference with section 1, ^$$^ p < 0.01 for difference with section 2, ^£££^p < 0.001 for difference with section 3.*

No significant correlation was found between the changes in general fatigue or peripheral pain, the flat equivalent speed and the parameters of posture.

## Discussion

PC kinetics showed a bi-phasic response. It increased until km 106 where all the standard parameters peaked. After this point, these parameters decreased until the end of the race where similar value at the PRE race are observed; revealing that standard parameters are regulated to control and minimize the alteration of PC. To our knowledge, this is the first study reporting such a pattern of PC adaptation during a MUM.

The kinetics of SDA, which appears to be more sensitive than standard parameters, showed a bi-phasic pattern, confirmed that the PC is more altered at km 106 and, particularly, in the AP plane. The critical point (inflexion point between short and long term regions of SDA) is moved up, meaning that the stochastic activity during the short-term region increased. This can reflect a disturbed proprioceptive feedback, increased reflex time, or an increase in agonist-antagonist co-activation (Degache et al., 2014). The fact that AP was more altered than ML might be explained by anatomical function. In a bipedal stance, PC is more stable in the frontal plane than the sagittal one, because AP is under ankle control (plantar/dorsiflexor), whereas ML is under hip control (abductor/adductor) due to anatomical joint orientations (Winter et al., 1996).

Compared to Degache et al. (2014) where no significant alterations of PC were found at mid race (151 km) compare to PRE condition, the present study revealed significant alterations at the same point, excepted for C*o*Py. This result could be explained by the fact that weather during the race was very difficult with lots of rain and very slippery trail, leading more muscle and joint fatigue in lower limbs, specifically about hip muscle. However, the current results suggest that peak alteration of PC appears relatively earlier during the MUM and before mid-race.

We could suppose that the results are in relation with the characteristic of MUM: the section 2 was the first and only one where the participants were running/walking through three pass over 3000 m, meaning that they spent more time at high altitude. The hypobaric hypoxia condition could have altered PC by means of different mechanisms such as a deficient in the vestibular system or a decrease in vision due to lack of oxygen (Nordahl et al., 1998; Degache et al., 2012). Change in barometric pressure seem to play a role in the inner ear and impact the vestibular nerve activity (Funakubo et al., 2010), either if the mechanism is not well describe hypobaric condition seem to impact PC more than hypoxic condition (Degache et al., 2012). A recent study on PC at moderate altitude showed that static control was impaired in an altitude dependent manner at 2590, 1630, and 490 m (Stadelmann et al., 2015). Moreover, it was demonstrated that a short exposure to hypobaric hypoxia at 3000 m impaired significantly PC parameters if compare to 400 m (*p* < 0.05) principally in the AP plane (Degache et al., 2012). An increase of the electromyographic activity of the plantar flexor was also observed in hypobaric hypoxia (Hoshikawa et al., 2010) It means that PC could be more impaired in the section where the runners were exposed to higher altitude.

Previous research also found adaptation and similar kinetics in T*d*G in order to counterbalance the extreme load and stress after the first half of the race. This kind of adaptive response was similar to Degache et al. (2016), which observed that the running pattern and spring mass behavior changed in the first half of the T*d*G. These changes were associated with nociceptive feedback and they consisted of a reduction of aerial time, ground reaction force (GRF), leg stiffness, and an increase in step frequency with the purpose to reduce pain (Degache et al., 2013). Saugy et al. (2013), revealed that general fatigue led to a decrease in speed during the second part of the race. Consequently this decrease in speed induced less muscle damage and inflammatory response. In relation with this study, after the first 50 km a large deceleration throughout the MUM is also observed, indicating that the running pattern probably changed and that safer pattern to reduce pain and to limit muscle damage appeared. The speed following the initial section until the last section is close to a walking speed. It was demonstrated that running generates greater damage for proprioception (muscles, tendon, joint), because it included stronger eccentric and concentric contraction than walking and involved more PC alterations (Paillard, 2012). We suppose that this decrease of speed influence indirectly the proprioceptive feedbacks of PC regulation and that walking is one of the adaptive responses.

A biphasic response in cardiac fatigue was also described during the same MUM and was explained by hemodynamics changes as the augmentation in extracellular water due to increased inflammation (Maufrais et al., 2016). These mechanisms might also interfere with PC changes. Indeed, Vitiello et al. (2015) reported a relationship between muscle swelling, increased hydric volume in the calves and peripheral fatigue in plantar flexors (PF) after the T*d*G. Since PF are important for PC (Vuillerme et al., 2002), peripheral oedema in the lower leg and fatigue of PF in MUM (Saugy et al., 2013) could impact the sensory response in the PC loop regulation.

General muscular exercise affects the musculoskeletal system by impairing the effectiveness of the postural regulating mechanisms (Lepers et al., 1997; Nardone et al., 1998). The process of deterioration of PC is multifactorial, some physiological effects are presented: (1) effect of the metabolic activation, (2) effect of intensity/duration of exercises, (3) effect of dehydration on the vestibular system, (4) disturbance of visual/vestibular information induced by running, and (5) disturbance of type of muscle contraction since PC is more disturbed after eccentric actions than concentric (Paillard, 2012). In T*d*G all these factors could appear because it includes duration, intensity and high positive and negative elevation.

In contrast of these sources of disturbance, compensatory strategies were presented in several studies (Vuillerme et al., 2002; Simoneau et al., 2006; Paillard, 2012). Sensory compensation by improvement of the sensory detection capabilities help the central nervous system to select the optimal balance control commands (Simoneau et al., 2006). It is also suggested that increased attentional demand seems to be more cognitively dependent during postural stability (Vuillerme et al., 2002).

Our results could be in link with the pacing strategy and the central governor model of exercise regulation, which suggests that during prolonged endurance exercise the central nervous system regulates exercise performance by continuously modifying the recruitment of motor unit, to insure that humans exercise with reserve and to ensure the whole body homeostasis (Swart et al., 2009; Noakes, 2012). All afferents information from each physiological system generate a conscious rate of perceived exertion managed by the brain. Consequently the output is adjusted in order to avoid the exercise termination and to preserve the participants (Saugy et al., 2013).

Sleep deprivation and fatigue, does not appear to significantly alter PC (lack of significant correlation), but still has an effect on the reduction of attention and sensorimotor integration (Degache et al., 2014). In connection with the respective feedback-feedforward of the pacing regulation, sleep deprivation and fatigue could explain the decrease in running/walking speed observed in our study, in order to limit the reduction of attention and reduce the risk of falls.

One limitation of this study is that it was not possible to analyze the PC during the entire race because of the inclement weather. The adverse weather, like coldness, may affect the PC parameters (Billot et al., 2013), by reducing the plantar sole sensitivity.

## Conclusion

The present study showed significant PC alterations in the first half of the race. The bi-phasic adaptation of PC, where values are returning near to baseline at km 200, confirmed that beyond the influence of exercise duration, the balance control system compensated the initial disturbance with pacing strategies and adaptive responses. Posture alteration is progressively increased until 100 km. After this point, compensatory mechanisms appear to limit the posture degradation.

## Author Contributions

GM, FD, FM, MF, and AS contributed to conception and design of the study. FD organized the database. FD, GM, ES, and KG performed the statistical analysis. FD and ES wrote the first draft of the manuscript. ES, GM, GV, FM, MF, and FD wrote sections of the manuscript. All authors contributed to manuscript revision, read and approved the submitted version.

## Conflict of Interest Statement

The authors declare that the research was conducted in the absence of any commercial or financial relationships that could be construed as a potential conflict of interest.
